# The Effect of Protein FAM172A on Proliferation in HepG2 Cells and Investigation of the Possible Molecular Mechanism

**DOI:** 10.1155/2019/5901083

**Published:** 2019-12-13

**Authors:** Hong Zhao, Yujie Wang, Yufeng Liu, Xiaohua Hao, Hongshan Wei, Wen Xie

**Affiliations:** ^1^Liver Diseases Center, Beijing Ditan Hospital, Capital Medical University, Beijing 100015, China; ^2^Department of Infectious Diseases, Peking University International Hospital, Beijing 102206, China; ^3^Institute of Infectious Diseases, Beijing Ditan Hospital, Capital Medical University, Beijing 100015, China

## Abstract

**Background:**

In our previous study, we found that the FAM172A recombinant protein could promote proliferation of L02 cells. However, the underlying mechanisms are still unknown. The present study was aimed at investigating the effect of FAM172A on proliferation of HepG2 cells and exploring the possible molecular mechanisms and its role in hepatocellular carcinoma (HCC).

**Methods:**

Cell proliferation was measured by MTT assay. Western blot test was carried out to investigate the mechanism. Rabbit antibodies against FAM172A and membrane proteins isolated from lysate of HepG2 cell were coprecipitated and the resultant precipitates were analyzed by mass spectrum.

**Results:**

The MTT assay showed that recombinant protein FAM172A isoform 1 (FAM172A-1) could induce HepG2 cell proliferation at the concentration of 10-100 ng/mL, while protein FAM172A isoform 3 (FAM172A-3) was at the concentration of 80-100 ng/mL. Western blot demonstrated that both FAM172A-1 and FAM172A-3 could activate the mitogen-activated protein kinase/extracellular signal-regulated protein kinase (MAPK/ERK) pathway and the phosphatidylinositol 3-kinase/threonine-protein kinase (PI3K/Akt) pathway. Mass spectrum analysis suggested that there were some membrane proteins interacting with FAM172A. Several candidate interacting proteins might mediate proliferation signals induced by FAM172A recombinant protein, including seven membrane proteins.

**Conclusion:**

In conclusion, FAM172A recombinant protein could induce proliferation of HepG2 cells, in which the MAPK/ERK and PI3K/Akt signaling pathways might be involved. The role of FAM172A in HepG2 cell proliferation also indicated its possible involvement in HCC. The receptor of FAM172A on cells still needs to be exploited.

## 1. Introduction

Liver cancer is the fourth among the most common causes of cancer deaths (782,000 deaths, 8.2%) as published by the International Agency for Research on Cancer in 2018 [1]. Among the primary liver cancers, the hepatocellular carcinoma (HCC) forms 85% to 90% of causes of cancer mortality. HCC can be prevented, detected at an early stage, and effectively treated with high-quality screening, proper management of screen-detected lesions, and appropriate therapy for the stage of disease [2]. The understanding of molecular mechanisms that induces hepatocarcinogenesis is growing. This risk of cancer is not existent in the healthy liver and is pronounced in response to chronic liver injury at the cirrhosis stage [3]. Gene expression profiling and proteomic analyses elucidated molecular events underlying HCC development and allowed to identify novel diagnostic markers as well as therapeutic and preventive targets [4]. Although disease at the advanced stage or with progression after locoregional therapy has a dismal prognosis and systemic therapy has not improved survival in patients with advanced hepatocellular carcinoma, sorafenib and lenvatinib are the drugs mostly used with little success at this stage [5, 6]. These data provide evidence that the pathogenesis and progression of HCC are mediated by a number of molecular defects and dysregulated pathways. Novel targeted therapies can be designed to inhibit these pathways at a molecular level in order to improve the clinical outcome [7, 8], and also, combination therapy that targets multiple different steps can be an appropriate strategy to combat human HCC [9]. The lethal nature of this cancer with high levels of genomic instability in advanced disease makes it necessary to identify biomarkers at the earliest disease stages that will aid in the development of successful therapeutic interventions [8, 10]. miRNA-517 and miR-122 are identified in HCC pathogenesis, tumor prognosis, and cell proliferation [11, 12]. Other pathways are mTOR activation and RICTOR oncogene as a mediator of human hepatocarcinogenesis [13]. Changes in the microenvironment play a major role in HCC and the overexpression of the MAPK kinase- (MEK-) MAPK in hepatocellular carcinoma, and change from a MAPK-independent cell survival pathway to a MAPK-dependent cell survival pathway is one among them [14–16]. Other pathways which can be targeted include the MAPK pathway (Ras/Raf/MEK/ERK), MEK, PI3K/Akt/mTOR pathway, VEGF/VEGFR, PDGFR, FGFR, EGF/EGFR, hepatocyte growth factor/c-Met pathway, and IGF/IGFR as listed by Klungboonkrong et al. [17]. Persistent hepatitis virus infection is also associated with HCC, and the MAPK/ERK cascade including Elk1 is involved in the process [18]. Li et al. has demonstrated that the FAM172A protein promoted cell proliferation, inhibited cell apoptosis, and facilitated S-phase entry and all these indicated that the FAM172A protein was involved in cell growth regulation [19]. FAM172A was identified as a new tumor-suppressor gene playing an important role in cell cycle control and tumor cell proliferation [20]. The FAM172A gene, also known as C5orf21, was first cloned from normal human aortic tissues in 2009. This gene is located on chromosome 5q15 and little is known about its function. Li et al. reported that the C5orf21 gene contained five splice variants and was identified for the first time at the level of mRNA while changes of C5orf21 gene expression are also correlated with diabetic macroangiopathy [21]. Li et al. presented evidence that the FAM172A protein existed in human aortic endothelial and human aortic smooth muscle cells and macrophages and had possible involvement in the pathogenesis of high glucose-induced vascular damage [22]. Li et al. showed that the FAM172A protein promoted the proliferation of human papillary thyroid carcinoma cells [23] while Cui et al. showed that FAM172A was a tumor suppressor in colorectal carcinoma [24].

In view of diagnosing HCC with biomarkers and treating HCC with target-specific drugs, in the present work, we investigated the effect of FAM172A on proliferation of HepG2 cells and explored the possible molecular mechanisms.

## 2. Materials and Methods

### 2.1. Reagents

Dulbecco's modified Eagle's medium (DMEM), penicillin, streptomycin, and fetal bovine serum were purchased from Hyclone (Logan, UT). MS-grade modified trypsin was from Promega (Madison, WI). Dimethylsulfoxide (DMSO) was purchased from Sigma (St. Louis, MO). Rabbit polyclonal antibodies against total MAPK/ERK, phospho-MAPK/ERK, total-Akt, phospho-Akt, and phosphatase inhibitor cocktail were obtained from Cell Signaling Technology (Boston, MA). 3-(4,5-Dimethylthiazol)-2,5-diphenyltetra-zolium bromide (MTT), aprotinin, and leupeptin were from Amresco (Solon, OH). A Mem-PER mammalian protein extraction reagent, Mem-PER eukaryotic membrane protein extraction reagent kit, and BCA protein assay kit were purchased from Thermo Fisher Scientific (Rockford, IL).

Protein A/G PLUS-Agarose Immunoprecipitation Reagent, monoclonal mouse antibody against human actin, or GAPDH was purchased from Santa Cruz Biotechnology (Santa Cruz, CA). Normal rabbit IgG was purchased from PeproTech (Rocky Hill, NJ). FAM172A recombinant proteins were preserved in our laboratory.

### 2.2. Cell Culture

All experiments were performed on the human hepatoma cell line HepG2 in this study. The cells were preserved in our laboratory. Cells were cultured in DMEM medium supplemented with 10% fetal bovine serum (FBS), penicillin, and streptomycin. Cultures were maintained under 95% humidified atmosphere containing 5% carbon dioxide at 37°C. Cells were passaged every other day at a 2 : 1 split ratio.

### 2.3. Proliferation Measurement

Cell proliferation was measured by the classic MTT assay [25]. In brief, HepG2 cells were seeded in 96-well plates (4000 cells/well) in 100 *μ*L culture medium with 10% FBS. After cell attachment for 12 h, the medium was replaced with a serum-starved medium (0.5% FBS) for 24 h. Then, cells were incubated with different concentrations of FAM172A-1 or FAM172A-3 recombinant protein (0, 1, 10, 20, 30, 40, 50, 80, and 100 ng/mL) for 24 h. Paralleling with the experiment, wells without cells served as the blank groups. Freshly prepared MTT solution (5 mg/mL of final concentration) was added to each well, and the cells were incubated for 4 h at 37°C. Then, the medium was aspirated carefully, and 150 *μ*L DMSO was added to each well to solubilize MTT. The optical density (OD) values were quantified in a Multimode Microplate Reader-Varioskan Flash at 490/570 nm.

### 2.4. Cell Lysate Preparation

Cells were seeded in 6-well plates. After 12 h, cells were serum-starved in medium with 0.5% FBS for 24 h. Then, FAM172A-1 or FAM172A-3 recombinant protein (100 ng/mL of final concentration) was added and cells were coincubated for 0 h, 4 h, 8 h, and 12 h. Equal volume medium served as the control. Subsequently, cells were washed with PBS and lysed in Mem-PER mammalian protein extraction reagent (protease inhibitor added beforehand) for 30 minutes on ice. Centrifugation was done at 12,000 g for 15 minutes at 4°C. The supernatants were collected and boiled in 1x sodium dodecyl sulfate sample buffer for 10 minutes at 99°C. The protein concentration was measured by BCA protein assay kit at 562 nm on a plate reader [26].

### 2.5. Western Blot Analysis

Proteins (50 *μ*g/lane) were separated on sodium dodecyl sulfate-polyacrylamide gel electrophoresis (SDS-AGE) and were transferred electrophoretically onto nitrocellulose membranes [24]. Then, membranes were blocked in 5% nonfat dried milk or 5% bovine serum albumin in buffer saline for 1 h at room temperature. Membranes were subsequently incubated with a specific primary antibody overnight at 4°C and then incubated with a corresponding horseradish peroxidase-conjugated secondary antibody for 1 h at room temperature. After each incubation, PBS washing was repeated 3 times. Proteins were eventually visualized with enhanced chemiluminescent reagents. *β*-Actin or GAPDH served as a control. Every experiment was repeated at least three times.

### 2.6. Preparation of Rabbit Polyclonal Antibody against FAM172A

Rabbit polyclonal antibody was prepared as described by Ren et al. and Zheng et al. with some modifications [27, 28]. Firstly, serum samples were collected before immunization. Then, rabbit was immunized with FAM172A-3 recombinant protein (1 mg) every other week for 3 times. For the first immunization, the FAM172A-3 recombinant protein was mixed with Freund's complete adjuvant and for the other immunizations with Freund's incomplete adjuvant. Serum samples were collected one week after the last immunization and the antibody was purified by a Protein A Sepharose column. The prepared polyclonal antibody was analyzed by ELISA and western blot, to ensure that the polyclonal antibody can be used in the following experiments.

### 2.7. Isolation of Membrane Protein

Cells (5 × 10^6^/mL) at 70% confluence were harvested and washed once by PBS. Membrane proteins were prepared using the commercial Mem-PER Eukaryotic Membrane Protein Extraction Reagent Kit as described by the manufacturer. The cell lysate mixture was centrifuged at 12,000 g for 15 minutes at 4°C. The supernatant was maintained and heated at 37°C for 20 minutes. Then, the supernatant was centrifuged at 10,000 g for 2 minutes at room temperature. The top phase was removed rapidly, and the bottom phase was a membrane protein. The identified membrane protein was normalized to the appropriate volume with Reagent B diluted 4-fold by ultrapure water.

### 2.8. Coimmunoprecipitation

The prepared membrane protein was divided into two, and each was normalized to 1 mL as described above. Normal rabbit IgG and protein A/G sepharose beads were added and incubated for 30 minutes at 4°C, followed by centrifugation at 2500 rpm for 5 minutes to remove the beads. The prepared polyclonal antibody or normal rabbit IgG (control group) was added in the supernatant and incubated for 1 h at 4°C with continuous rotation. Then, protein A/G sepharose beads were added and incubated for 4 h at 4°C with continuous rotation. Centrifugation was done at 2500 rpm for 5 minutes and the supernatant was removed. After washing the beads, proteins were eluted from the beads in a 1x SDS sample buffer and boiled for 10 minutes at 99°C. The precipitates were separated by SDS-PAGE and stained with Coomassie Brilliant Blue as discussed [29].

### 2.9. Mass Spectrometric Analysis

The targeted gel bands were cut into small pieces and underwent in-gel tryptic digestion and were prepared for mass spectra analysis as previously described [30]. The gels were destained with 50% CH_3_CN, 30% CH_3_CN/100 mM NH_4_HCO_3_, 50% CH_3_CN/50 mM NH_4_HCO_3_, and 50% CH_3_CN/25 mM NH_4_HCO_3_ one by one, until the gels became clarified. After drying, the gels were digested with MS-grade modified trypsin for 1 h at 4°C. Then, add sufficient volume of 25 mM NH_4_HCO_3_ to cover the gel pieces and continue to digest for 16 h at 37°C. 50 *μ*L double-distilled H_2_O was added and incubated for 10 minutes. Peptides were extracted with 60% CH_3_CN/5% TFA from the gel pieces and dried to 10 *μ*L volume. The prepared peptides were then identified on a Q-TOF mass spectrometer, and data were analyzed with the Mascot search software.

### 2.10. Statistical Analysis

The quantitative data were expressed as mean ± SD. Statistical analysis was performed with GraphPad Prism 5.01 using ANOVA analysis followed by Tukey post hoc test. The *p* value less than 0.05 was considered statistically significant.

## 3. Results

### 3.1. FAM172A Promoted HepG2 Cell Proliferation

**Figure 1:**
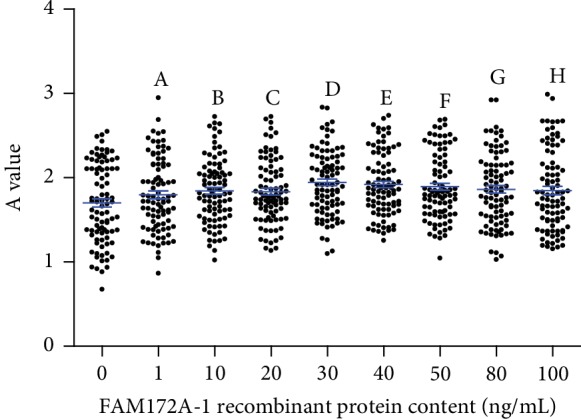
The effect of FAM172A-1 recombinant protein on cell proliferation in HepG2 cells. After being serum-starved for 24 h, HepG2 cells were treated with the indicated concentration of FAM172A-1 recombinant protein for 24 h. Cell proliferation was measured by MTT assay for four independent experiments. Comparing with the control group, the 10-100 ng/mL (B–H) groups were significantly different (*p* < 0.05).

**Figure 2:**
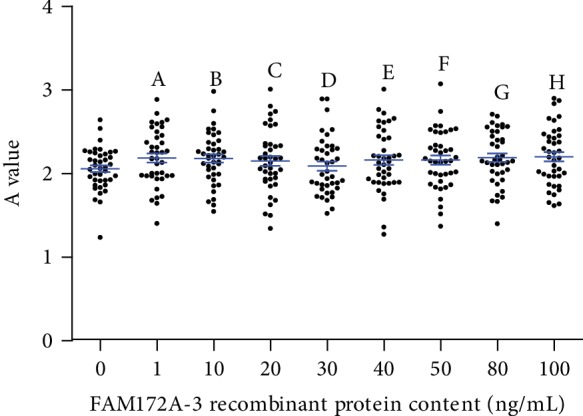
The effect of FAM172A-3 recombinant protein on cell proliferation in HepG2 cells. After being serum-starved for 24 h, HepG2 cells were treated with the indicated concentration of FAM172A-3 recombinant protein for 24 h. Cell proliferation was measured by MTT assay for four independent experiments. Comparing with the control group, only the 80 ng/mL and 100 ng/mL (G, H) groups were significantly different (*p* < 0.05).

After incubation with FAM172A-1 or FAM172A-3 recombinant protein for 24 h at the indicated concentrations, the proliferation of HepG2 cells was measured by MTT assay. We found that cell proliferation was promoted by FAM172A recombinant protein. At the concentration of 1 ng/mL, FAM172A-1 showed no significant effect on cell proliferation (*p* > 0.05), while at 10-100 ng/mL incubation concentration, the levels of cell proliferation were significantly higher than those of the control (*p* < 0.05; Figure 1). However, the proliferation effects were only observed at the incubation concentration of 80 ng/mL and 100 ng/mL, when the FAM172A-3 recombinant protein was cocultured (*p* < 0.05; Figure 2).

### 3.2. MAPK/ERK and PI3K/Akt Signal Pathway Might Be Involved

**Figure 3:**
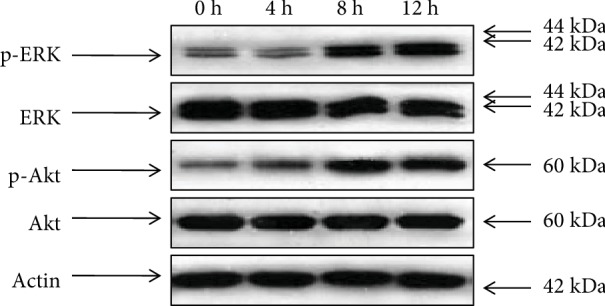
FAM172A-1 recombinant protein activated MAPK/ERK and PI3K/Akt pathways in HepG2 cells. After being serum-starved for 24 h, HepG2 cells were treated with FAM172A-1 recombinant protein (100 ng/mL) for 0 h, 4 h, 8 h, and 12 h. Western blot analysis showed that the level of ERK1/2 and Akt phosphorylation was significantly increased after treatment with FAM172A-1 while the level of total ERK1/2 and Akt remained unchanged.

**Figure 4:**
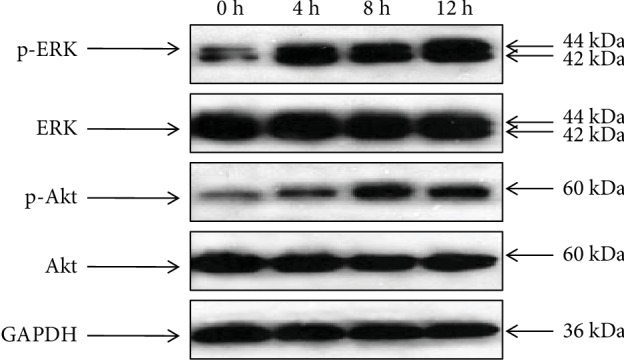
FAM172A-3 recombinant protein activated MAPK/ERK and PI3K/Akt pathways in HepG2 cells. After being serum-starved for 24 h, HepG2 cells were treated with FAM172A-3 recombinant protein (100 ng/mL) for 0 h, 4 h, 8 h, and 12 h. Western blot analysis showed that the level of ERK1/2 and Akt phosphorylation was significantly increased after treatment with FAM172A-3 while the level of total ERK1/2 and Akt remained unchanged.

To explore the molecular mechanism by which the FAM172A protein promoted cell proliferation, we studied the effect of FAM172A-1 or FAM172A-3 on two pivotal signal pathways, which were closely associated with the cell proliferation of HepG2 cells. The cells were stimulated by the FAM172A-1 or the FAM172A-3 recombinant protein, and the activities of MAPK/ERK and PI3K/Akt were analyzed. The results showed that the level of ERK1/2 and Akt phosphorylation was significantly increased after treatment with the FAM172A recombinant protein while the level of total ERK1/2 and Akt remained unchanged (Figures 3 and 4).

### 3.3. Activity of Rabbit Polyclonal Antibody against FAM172A

**Figure 5:**
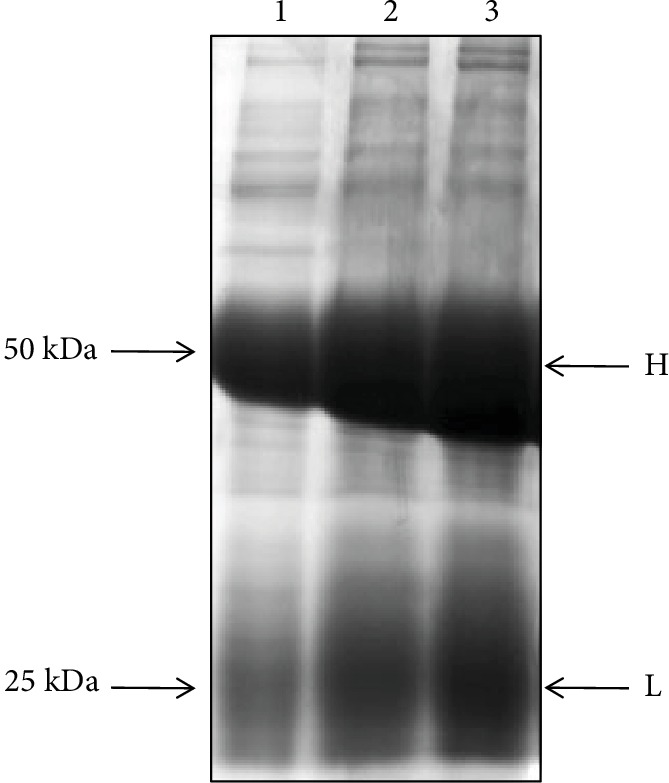
Purification of rabbit anti-human polyclonal antibody. The prepared antibody had a good purity. H: heavy chain; L: light chain.

**Figure 6:**
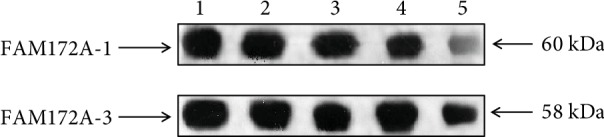
Identification of recombinant protein using the prepared polyclonal antibody. The prepared antibody had a good specificity. Lanes1-5 represented different antibody dilution ratios: 1 : 5000, 1 : 10000, 1 : 20000, 1 : 40000, and 1 : 80000, respectively.

Results showed that the prepared antibody had a high purity (Figure 5) and a good specificity (Figure 6).

### 3.4. Identification of Membrane Proteins Interacting with FAM172A

**Figure 7:**
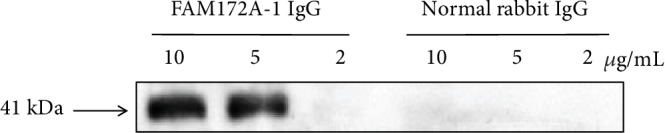
Exploration of the best antibody concentration in coimmunoprecipitation. Different concentrations of polyclonal antibody (2 *μ*g/mL, 5 *μ*g/mL, and 10 *μ*g/mL) were used in the coimmunoprecipitation experiment; meanwhile, the corresponding concentration of normal rabbit IgG served as control groups. Western blot analysis indicated that 5 *μ*g/mL was the best condition.

**Figure 8:**
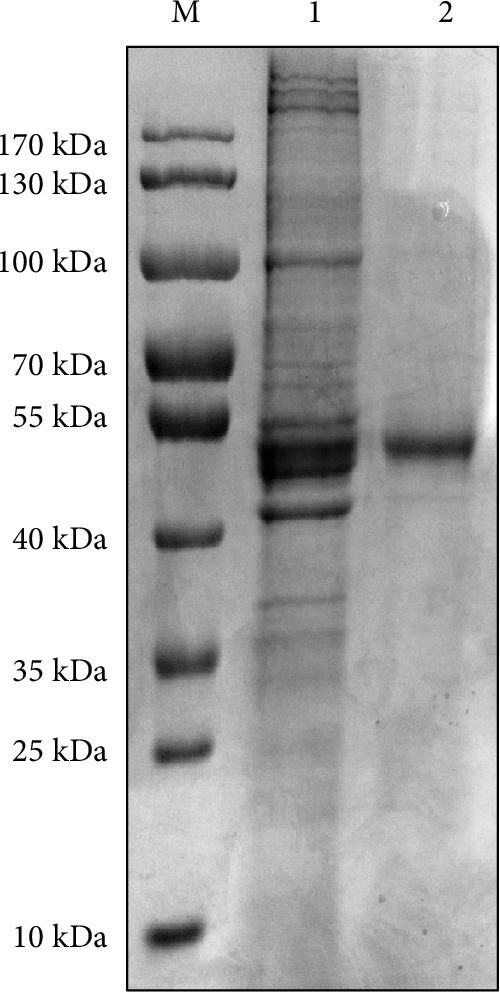
Separated precipitates by SDS-PAGE. Lane 1: precipitates pulled down by rabbit polyclonal antibody against human FAM172A recombinant protein; lane 2: precipitates pulled down by normal rabbit IgG.

Firstly, we explored the best concentration of rabbit polyclonal antibody against FAM172A in coimmunoprecipitation. Different concentrations (2, 5, and 10 *μ*g/mL) were used in the coimmunoprecipitation experiment, and the precipitates were analyzed by western blot. Western blot analysis showed that 5 *μ*g/mL was the best concentration of the FAM172A antibody when compared to normal rabbit IgG (Figure 7). With the best concentration, the coimmunoprecipitation experiment was carried out and the precipitates were analyzed by SDS-PAGE. The results showed that there were several bands significantly different with the control (Figure 8). Then, those differential protein bands were cut down and tryptic digested in-gel. The well-digested peptides were identified on an UPLC-Q-TOF mass spectrometer. Mascot search results showed that there were seven membrane proteins which might interact with FAM172A (Table 1). The other twelve identified proteins were not membrane proteins, or the subcellular locations had not been confirmed (Table 2).

## 4. Discussions

There are four FAM172A transcript variants. The resulting isform 1 has the most complete structure and is a kind of secretory protein, which makes it the most potential one to regulate cell growth. The resulting isoform 2 has a shorter N-terminus, compared to isoform 1. The resulting isoform 3 has a shorter N-terminus and lacks an internal segment, compared to isoform 1. The variant 4 lacks two alternate exons, compared to variant 1. This variant is represented as noncoding because the use of the 5′-most-supported translational start codon renders it a candidate for nonsense-mediated mRNA decay. So we selected FAM172A-1 and FAM172A-3 isoforms in our experiment and found their involvement in HepG2 cell proliferation. Firstly, the effective concentrations of FAM172A-1 and FAM172A-3 were identified, which lead to HepG2 cell proliferation, by coculturing the proteins and measuring with the MTT assay. Our results were in accordance with other studies published where FAM172A was significantly upregulated and was found to have active involvement in the cell growth cycle. In a study by Cui et al., FAM172A expression was associated with the malignant degree of the tumor which could be used to identify colorectal cancer patients with a more serious disease stage for appropriate early treatment. Cui et al. has drawn a correlation between the circulating tumor cell positive rate and the FAM172A expression positive rate [31]. Another study by Li et al. on the effect of the FAM172A protein on HEK293 cells demonstrated similar results. The FAM172A protein promoted cell proliferation, inhibited cell apoptosis, and facilitated S-phase entry, indicating its involvement in cell growth regulation and possible role in diabetic macroangiopathy [19].

We next tried to identify the possible molecular mechanisms of the FAM172A protein promoting HepG2 cell proliferation. Although a number of pathways are involved in HCC [17], two crucial signal pathways, MAPK/ERK and PI3K/Akt, closely associated with the cell proliferation [32] of HepG2 cells, were studied by the in vitro cell culture method. HepG2 cells cocultured with FAM172A proteins were lysed and the western blot test carried out. Results revealed that the expression of FAM172A was upregulated and the level of ERK1/2 and Akt phosphorylation was significantly increased after treatment with the FAM172A recombinant protein while the level of total ERK1/2 and Akt remained unchanged. This indicated the involvement of the MAPK/ERK and PI3K/Akt pathways. Consistent with these results were the work by Li et al. which investigated the effect of FAM172A on several common signaling pathways associated with cell proliferation and demonstrated that overexpression of FAM172A caused marked activation of the p38 MAPK pathway inducing cell proliferation. Another study also showed the involvement of FAM172A and the p38 MAPK pathway in the disease course of papillary thyroid carcinoma [23]. Contrastingly, other studies have reported that FAM172A was a tumor-suppressor gene which mediated cell cycle arrest at least partially, by upregulated expression of the Notch 3 pathway [20]. Cui et al. has also reported in a work with a colorectal cancer model that FAM172A might inhibit the growth and invasion of colorectal cancer cells and the mechanism involved activation of ERS signaling [24]. Qian et al. demonstrated that FAM172A was upregulated by the STAT1 transcription factor and also downregulated, thus modulating apoptosis and the proliferation process of colon cancer cells (human LoVo and SW480 cells) [33]. Similar in line with the results in our studies, Wang and Tai demonstrated that high expression levels of MAPK, phosphorylated MAPK, and phosphorylated MEK1/2 were found in tumors of HCC patients [16]. Although there were many other pathways involved in cell growth that we have not comprehensively screened and secondary effects could not be excluded, our results showed that MAPK/ERK and PI3K/Akt signaling pathways were likely to be involved. Maybe we can block either pathway for further validation.

Next in our study, we isolated a high-purity and good specificity rabbit polyclonal antibody against the recombinant FAM172A. The best concentration of these antibodies was coimmunoprecipitated with isolated membrane proteins obtained from the HepG2 cell lysate, and the precipitates were separated by SDS-PAGE electrophoresis. Differential protein bands were identified on an UPLC-Q-TOF mass spectrometer. Seven membrane proteins and twelve nonmembrane proteins were identified interacting with FAM172A. The membrane proteins were MACF, PTPRF, NDST, SLC27A6, ACVRL1, RAB27A, and RAB3D identified as binding with FAM172A leading to proliferation of the HepG2 cell by the upregulation or overexpression of these proteins. Our results are consistent with other published data [34–38]. Further studies need to validate these candidate interacting membrane proteins of FAM172A, including Co-IP, western blot, antibody blocking, siRNA technology, and histological verification.

## 5. Conclusion

In conclusion, the strength of this study lies in determining the possible mechanism of FAM172A for proliferation of HepG2 cells. Both MAPK/ERK and PI3K/Akt signaling pathways might be involve. The membrane proteins and nonmembrane proteins involved in the cascade of MAPK/ERK and PI3K/Akt were also detected. This would go a long way in HCC treatment for the synthesis of diagnostic markers and target drugs. The limitation of this study is that the receptors of FAM172A on cells still need to be exploited.

## Figures and Tables

**Table 1:** Membrane proteins possibly interacting with FAM172A.

Mw	Protein name	Official symbol	GI no.
843	Microtubule-actin cross-linking factor 1	MACF1	48734767
214	Receptor-type tyrosine-protein phosphatase F	PTPRF	226709091
101	Bifunctional heparan sulfate N-deacetylase/N-sulfotransferase 4	NDST4	86577748
71	Long-chain fatty acid transport protein 6	SLC27A6	48146375
57	Serine/threonine-protein kinase receptor R3	ACVRL1	3915750
25	Ras-related protein Rab-27A	RAB27A	1931577
24	Ras-related protein Rab-3D	RAB3D	20379042

**Table 2:** Other proteins possibly interacting with FAM172A.

Mw	Protein name	Official symbol	GI no.
112	Protein FAM179A	FAM179A	172046176
116	PHD finger protein 20-like protein 1	PHF20L1	317373307
85	Zinc finger protein 280C	ZNF280C	74751215
82	FAST kinase domain-containing protein 2	FASTKD2	74734717
62	Zinc finger protein 324B	ZNF324B	74757410
51	Homogentisate 1,2-dioxygenase	HGD	296434531
49	Ras association domain-containing protein 8	RASSF8	68068057
49	Probable tubulin polyglutamylase TTLL1	TTLL1	20455347
48	Zinc finger and SCAN domain-containing protein 31	ZSCAN31	23396994
38	Xin actin-binding repeat-containing protein 2	XIRP2	166919144
36	Calponin-3	CNN3	6225157
22	Protein SSX9	SSX9	74749935

## Data Availability

The data used to support the findings of this study are included within the article.
